# Different Faces for Different Places: Heterogeneity of Neutrophil Phenotype and Function

**DOI:** 10.1155/2019/8016254

**Published:** 2019-02-28

**Authors:** Peiqing Yang, Yanhong Li, Yan Xie, Yi Liu

**Affiliations:** Department of Rheumatology, West China Hospital, Sichuan University, Guoxue Xiang 37, Chengdu, Sichuan 610041, China

## Abstract

As the most abundant leukocytes in the circulation, neutrophils are committed to innate and adaptive immune effector function to protect the human body. They are capable of killing intruding microbes through various ways including phagocytosis, release of granules, and formation of extracellular traps. Recent research has revealed that neutrophils are heterogeneous in phenotype and function and can display outstanding plasticity in both homeostatic and disease states. The great flexibility and elasticity arm neutrophils with important regulatory and controlling functions in various disease states such as autoimmunity and inflammation as well as cancer. Hence, this review will focus on recent literature describing neutrophils' variable and diverse phenotypes and functions in different contexts.

## 1. Introduction

Neutrophils, considered as infantrymen in the innate immune system, are indispensable in safeguarding the human body against encroaching microbes. Generated from the bone marrow and circulating in the blood, neutrophils are a critical “second defense” standing behind the skin and mucus. As precursor leukocytes to be enlisted in inflammatory sites [1], neutrophils possess the capacity of both intra- and extracellular mechanisms [2, 3] to eliminate pathogens. Their very singular features, such as short lifespan and lower transcriptional activity, have led to the overly simplistic perception that neutrophils are homogenous with limited phenotypic heterogeneity. However, this classical view has been greatly challenged since different phenotypes have been reported in both healthy and pathologic conditions. Are all neutrophils generated equally? Do they share the same phenotypes in different environments? For those who know the Face Changing Dance of the traditional Chinese Sichuan Opera, you may appreciate the dance as a metaphor for the heterogeneity of neutrophil phenotype and function. Just like the performance of face changing in the Chinese Sichuan Opera, neutrophils resemble the actors expressing “different faces” in different conditions and places. We describe the elasticity of neutrophils and discuss their multiple phenotypes and functions.

## 2. Growth Footprint of Neutrophils

As the major activity of the bone marrow, almost two-thirds of the hematopoiesis is dedicated to myelopoiesis [2], and around 1 to 2 × 10^11^ neutrophils are generated every day. Granulopoiesis is under the control of multiple physiological and environmental cues. The feedback loop of IL-23, IL-17A, and granulocyte colony-stimulating growth factor (G-CSF) is vital to the regulation of granulopoiesis. Phagocytosis of apoptotic neutrophils by macrophage and dendritic cells depresses their production of IL-23, thus reducing IL-17A production by T cells and neutrophils, which leads to the downregulation and reduced production of G-CSF by fibroblasts and epithelial cells and reduction in neutrophil generation [4–6]. By contrast, the upregulation of G-CSF increases granulopoiesis and triggers chemokine receptor type 2 (CXCR2) signaling and neutrophil release [7, 8].

There are six stages in neutrophil maturation: myeloblast, promyelocyte, myelocyte, metamyelocyte, band cell, and polymorphonuclear [2], during which the transcription factors C/EBP*α* (CCAAT/enhancer-binding protein *α*) and ETS (E26 transformation specific) family transcription factor PU.1 (Spi-1) play fundamental roles. The balance between the two factors maintains a precise state of myeloid lineage commitment to granulocyte or monocyte. PU.1 is essential for monocyte differentiation while C/EBP*α* promotes granulocyte differentiation [9–14]. Other transcription factors including Lef-1, Gfi-1, and C/EBP*ε* are also conductive to terminal granulopoiesis [15–20].

## 3. Circulating Neutrophils: Fresh and Aged

**Figure 1:**
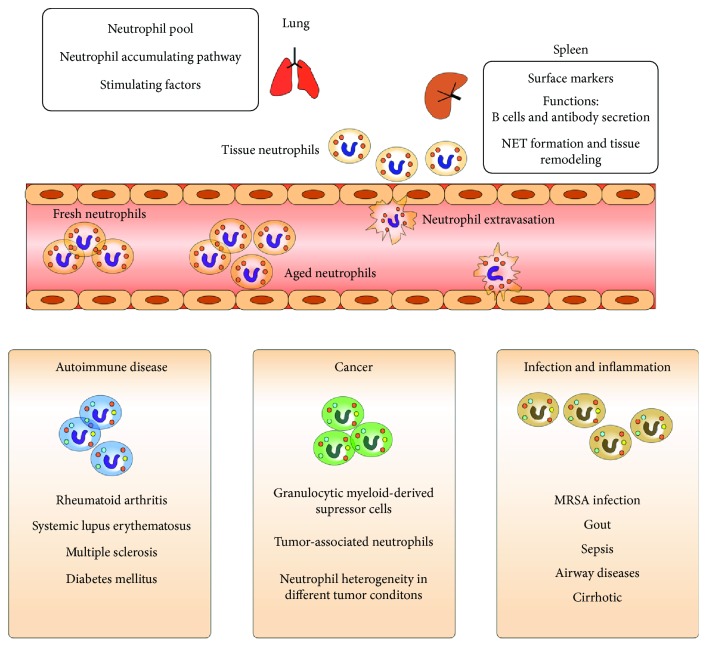
Heterogeneity of neutrophils in both health and disease. Neutrophils protect human body from intruding microbes and they display notable heterogeneity in blood circulation and specific tissues. After being activated by pathogens, neutrophils extravasate from the blood vessel and function as “immune soldiers” in various states. Intriguingly, neutrophils are verified to share multiple phenotypes and functions in autoimmune disease and cancer as well as inflammation and infection.

Though neutrophils have a half-life of only a few hours in the circulation, they nonetheless achieve phenotypic heterogeneity before migrating into tissues (Figure 1). It has been demonstrated *in vivo* that during a four-hour circulation in peripheral blood from the beginning of release from the bone marrow to clearance by macrophages, neutrophils change phenotype and morphology. This progression from fresh, new bone marrow emigrants to aged neutrophils and total number of neutrophils is regulated in a circadian way [21, 22].

CXCR4 and CXCR2 play vital roles in neutrophil retention in the bone marrow. WHIM syndrome (warts, hypogammaglobulinemia, infections, and myelokathexis) is clinically characterized by the overaccumulation of neutrophils in the bone marrow, which can be linked to a mutation of CXCR4 [23]. Deletion of CXCR4 or CXCR2 has a similar negative effect on neutrophil migration from the bone marrow to circulation [8, 24]. Neutrophils isolated from fresh blood have upregulated CXCR4 expression after four hours' culture *in vivo* [25]. Higher expression of CXCR4 combined with lower expression of CD62L promotes longer residency of neutrophils in circulation *in vivo* [21].

As for the aged neutrophils, some membrane molecules are increased including CD11b (*α*M) and CD49d (*α*4), the alpha subunits for integrins Mac-1 (macrophage-1 antigen, also known as *α*_M_*β*_2_), VLA4 (very late antigen 4, also known as integrin *α*4), TLR4 (Toll-like receptor 4), ICAM-1 (intercellular adhesion molecule-1), CD11c, CD24, and CD45 [26] while the expression of CD47, which promotes resistance to phagocytosis by macrophages, is downregulated [27]. The classical TLR4 agonist lipopolysaccharide (LPS) induced age-dependent changes in human neonatal neutrophil migration, gene expression, and cytokine production [28]. In addition to the aforementioned molecular changes, morphological alterations appear as smaller size, fewer granules, and a more multilobular nucleus in aged cells [21]. Signaling pathways related to cell activation, pathogen recognition, cell adhesion, migration, and apoptosis are modified in aged cells [27]. All of these changes assist mature circulating neutrophil migration to inflamed tissues and are consistent with similar changes in sites of inflammation [22].

## 4. Neutrophil Extravasation: Go to the Battlefront

### 4.1. Rolling

The very distinguished feature of neutrophil extravasation starts from the activation of endothelial cells. There are two ways to initiate this process: endothelial cells can be directly stimulated by surrounding pathogens or indirectly, irritated by inflammatory mediators released from resident leukocytes like tumor necrosis factor *α* (TNF-*α*), IL-1 and IL-17 [1, 29, 30]. Subsequent changes take place on the luminal surface where the expression of E-selectin, P-selectin, and *α* and *β* integrins is upregulated. Binding between cell surface glycoproteins such as P-selectin ligand 1 (PSGL-1) and P-selectin helps capture free neutrophils to the endothelial surface. E-selectin's binding with E-selectin ligand 1 (ESL-1) helps slow neutrophil rolling speed, and binding with CD44 leads to a distribution change of PSGL-1 and L-selectin, which also contributes to further reduction of rolling speed [31].

### 4.2. Adhesion

When it comes to the process of firm adhesion, *β*2 integrins LFA-1 (lymphocyte function-associated antigen 1, also known as *α*_L_*β*_2_) and MAC-1, together with their ligands ICAM-1 and ICAM-2 expressed on endothelial cells, are key molecules. LFA-1 binding to ICAM-1 initiates a change in neutrophil motion from rolling to adhesion [32], while the activation of LFA-1 depends on signals from PSGL-1 and CD44 [33]. The activation of G protein-coupled chemokine receptors on neutrophils leads to the conformational change of cell surface integrins that subsequently show higher affinity for their ligands. Overall, the link between integrins and ligands strengthens outside-in signaling pathways in neutrophils, reinforcing adhesion and initiating cell motility [1].

### 4.3. Crawling

When neutrophils set about migrating across endothelial cell-cell junctions, it is necessary for them to crawl effectively. Considering the existence of shear force in the blood flow, crawling vertically would be the best way to move to the endothelial junction. Neutrophils are endowed with the capacity to crawl forward while retaining the adhesion to the endothelial surface. Actin-binding proteins such as mammalian actin-binding protein 1 (MABP1, also known as drebrin-like protein) fortify the high-affinity conformation of *β*2 integrins to strengthen the interactions with actin, helping neutrophils crawl stably in the shear condition of blood flow. Additional signaling by VAV1 (a guanine exchange factor for the RHO-family GTPase RAC) and CDC42 (cell division control protein 42, a major regulator of organization of the actin cytoskeleton during leukocyte polarization and migration) contributes to neutrophil crawling [34] although the detailed mechanism is still poorly understood.

### 4.4. Migration

Particular types of migration include transmigration, abluminal crawling, and interstitial migration to inflamed foci. Transmigration requires integrins and CAMs (ICAM-1, ICAM-2, and VCAM-1 (vascular cell adhesion protein 1)) as well as various junctional proteins, such as CD31, CD99, CD155, and CD157 (reviewed in reference [1]). There are two ways for neutrophils to pass through the endothelium: paracellular and transcellular. Most neutrophils use paracellular migration, which is more efficient and takes shorter time (about 2-5 minutes) [1]. In transcellular migration, endothelial cells form microvilli-like transmigrating cups, which are projections enriched in ICAM-1 and VCAM-1, and form cuplike structures around adherent neutrophils in an LFA1- and VLA4-dependent manner [35, 36]. What is noticeable is that transcellular migration differs from phagocytosis, as the neutrophils *ipso facto* never enter the intracellular compartment of the endothelial cell.

The endothelial basement membrane consists of extracellular matrix containing collagens and laminins. Proteases in granules (described in detail below) vest neutrophils with the ability to break down the matrix, such as elastase (azurophil granules), matrix metalloproteinase 8 (MMP8) (specific granules), MMP9, and the membrane-attached matrix metalloproteinase MT6-MMP (gelatinase granules and secretory vesicles) [37].

Rab small GTPases (guanosine triphosphatases) belong to the Ras superfamily and are constituted of around 60 family members in mammals. Rab family GTPases play crucial roles in intracellular membrane trafficking, including cargo sorting, vesicle budding, vesicle formation, vesicle transport, docking, tethering, and fusion of vesicles with target membranes in eukaryotic cells [38]. Rab27, a Rab subfamily member which is known to control granule exocytosis, has two main isoforms: Rab27a and Rab27b. Rab27 stimulates elastase release from azurophilic granules, thus allowing local proteolysis of the extracellular domain of CD11b leading to uropod detachment and forward movement of the cell [39, 40]. Neutrophil proteases and its intracellular transport system provide the means for neutrophils to move through the extracellular matrix, but the complete mechanism for neutrophil extravasation requires more detailed understanding.

## 5. Tissue-Residing Neutrophils: One or More?

Recruitment and migration to tissues and organs are necessary processes for neutrophil functions [41]. Pattern recognition receptors (PRRs) expressed in local epithelial, endothelial, and dendritic cells, for example, such as TLRs and NOD-like receptors (NLRs), can be triggered in both infected and noninfected conditions. That activation may lead to increased vascular permeability and the release of chemokines to enhance neutrophil recruitment to the infected sites [1, 42]. So, neutrophils that have migrated into tissues are more active as phagocytic cells than blood neutrophils. Do all of extravasated neutrophils share a similar phenotype, or are they *per se* heterogeneous? Recent evidence is supportive for multiple phenotypes among tissue-resident neutrophils in different organs and sites.

In contrast to other organs, the lung is outstanding because of the high numbers of neutrophils that accumulate in pulmonary vessels. *β*2-integrins and very late antigen 4 (VLA4) are necessary for neutrophil adhesion, transmigration, and diapedesis into the lung tissue [1]. The smaller diameter of capillary segments (around 5 *μ*m) enhances extravasation of neutrophils by strengthening vessel wall contacts since the diameter of a neutrophil is 7-8 *μ*m. On the other hand, that change shows a high demand for cell deformation [43, 44], which is regulated by multiple factors, including anaphylatoxin C5a, chemotactic tripeptide fMLF (N-formyl-Met-Leu-Phe) [45–48], and bacterial compounds such as LPS [45, 49]. Neutrophils that collect in vascular lumen and interstitial space are maintained via a CXCR4-dependent pathway [50]. Since CXCR4 is expressed on aged neutrophils, it is possible that they may be the first class of neutrophils to migrate into the lung tissue [22], so the lungs may be a pool for primed neutrophils.

In the splenic marginal zone, neutrophils are capable of producing various cytokines, particularly IL-21, BAFF, APRIL, and TNF, to promote B cell differentiation and antibody production [51]. They are CD62L^low^ CD11b^hi^ICAM-1^hi^ and also assist in the formation of neutrophil extracellular traps (NETs, described in detail below) [52]. The neutrophils in draining lymph nodes play a direct role in phagocytic killing and also protect against pathogens via noncell autonomous mechanisms including the release of NETs, producing matrix metalloproteases and participating in tissue remodeling [53], but the further impact of macrophages removed by neutrophils remains unknown. It has been well studied that CXCR4 is necessary for the migration of neutrophils to lymph nodes, but the function of CCR7 is controversial since neutrophils could migrate to draining lymph nodes through a CCR7-independent way following *S. aureus* infection [54]. Besides, the *β*2 integrin CD11b also plays an important role in neutrophil migration from lymph vessels to lymph nodes [55], but the precise ligand has yet to be unambiguously determined [56].

## 6. Neutrophil Clearance and Reversed Migration

Tissue-resident neutrophils undergoing apoptosis are finally removed by neighboring macrophages and dendritic cells via phagocytosis. This process forms a feedback loop to control the production of neutrophils in the bone marrow [6]. Neutrophils in inflamed tissue are traditionally considered to be phagocytosed by macrophages; however, other research has indicated that extravasated neutrophils may reenter the circulation [57–59]. These reverse migrating neutrophils are considered to migrate to remote infected tissues to kill microbes, which makes effective utilization of neutrophils' capacity to fight infection [1].

## 7. Granule Biogenesis and Heterogeneity

Granules are the notable features of neutrophils containing various proteins to kill phagocytosed pathogens and digest damaged tissues. There exists a continuum in granules manifesting as four unique subsets: primary (azurophilic) granules, containing myeloperoxidase (MPO) and azurocidin; secondary (specific) granules, containing lactoferrin; tertiary (gelatinase) granules, containing MMP9 (gelatinase B); and secretory vesicles. The granule subsets are formed in a consecutive manner during granulopoiesis [60–62]. Azurophil granules are formed at the promyelocyte stage; the other granule subsets are formed during the myelocyte to segmented cell stage differentiation. The chronologic heterogeneity of granulopoiesis can be clarified by a mechanism called “targeting by timing of biosynthesis” [63], and the formation of granule proteins is confined to relevant stages of myelopoiesis. Intriguingly, the timing of granule subtype synthesis is not entirely concomitant with the expression of granule protein, which results in heterogeneity within granules of the same granule subtype [62].

In general terms, granule heterogeneity is revealed by neutrophil function and activity. Primary granules accumulate multiple antibacterial proteins such as MPO, azurophil, cathepsin G, elastase and proteinase 3, lysozyme [64, 65], enzymatic inactive CAP37 (protease cationic antimicrobial protein of 37 kDa) (azurocidin) [66], NSP4 (neutrophil serine protease 4) [67], and defensins. The heterogeneity of azurophil granules exists in both protein content and subcellular targeting [62]. Some granules are transported to the cell surface while others fuse with phagosomes. A vital member of the Rab GTPases family, Rab27, along with its effector, mammalian uncoordinated 13-4 (Munc13-4), regulates the targeting of the granules. Interaction between Rab27a and Munc13-4 mediates degranulation of neutrophils [68–70]. Secretory granules express Rab27 and fuse with the cell membrane via a Munc13-4-dependent mechanism, while nonsecretory granules fuse with phagosomes by a Munc13-4-independent mechanism. These mechanisms might be associated with changes in concentration and types of proteins contained in the fusing granules. It has been reported that nonsecretory granules display a high concentration of antibacterial proteins, 500 mg/ml in phagosomes [71]. Some azurophil granules are defensin-poor [72, 73], and the amount of defensin also varies. Some classical membrane proteins of lysosomes, such as LAMP (lysosome-associated membrane proteins) I and II, are not expressed on azurophil granules [74], which is inconsistent with the view that azurophil granules are specialized lysosomes though LAMP III is expressed on azurophil granules [65, 75, 76].

Secondary and tertiary granules have important roles in neutrophil extravasation and migration, and they display a morphological and functional continuum rather than discrete subsets [65]. There are three combinations of surface markers that define granule subsets. Specific granules are lactoferrin+/gelatinase−; gelatinase granules are lactoferrin-/gelatinase+; and “hybrid” granules containing both features are lactoferrin+/gelatinase+. Although these granules contain similar proteins, they play distinct roles in the anti-infection process. Gelatinase granules, containing less antibacterial proteins like lactoferrin, are exocytosed earlier than the other two kinds of granules followed by the hybrid granules, and specific granules are exocytosed last to kill microbes [77]. The chronology of granule exocytosis is consistent with granules' functions: when tissues are invaded by pathogens, antimicrobial granules are released after those that aid migration. Rab GTPases seem to play an important role in this process. The trafficking of specific and gelatinase granules to the cellular membrane is via the Rab27- and Munc13-4-dependent pathway [70]. Rab32 cycles with Rab38 and regulates the endosomal trafficking system (reviewed in reference [78]). Rab32 has not only been shown to be associated with the biogenesis of lysosome-related organelles, such as melanosomes, but also in controlling intracellular pathogens. For example, Rab32 has also been shown to be involved in the degradation of intracellular *Listeria monocytogenes* by enclosing the listeria that escapes from listeria-containing vesicles into the cytoplasm [79]. Far more understanding of the Rab32-mediated host defense mechanism should be a focus for both cell biology and medical research. Granule defects often lead to diseases, for example, granules that are abnormally clustered and polarized participate in the development of specific granule deficiency (SGD). Besides, the abnormally clustered granules also express proteins with limited glycol-epitopes [80], displaying abnormality in function.

Secretory vesicles (SVs) are formed in the late stage of neutrophil phenotypic progression and function to convey membrane proteins to the cell surface. The proteins packaged in SVs include the formyl peptide receptor 1 (fPR1), the integrin Mac-1 (*α*M*β*2), the phagocytic receptors CD16, together with CXCR2, which are vital for extravasation and migration [65]. These proteins enhance the expression of integrins and chemotactic receptors on neutrophil membrane and promotes neutrophil extravasation and function in inflammatory tissues.

## 8. Neutrophil Heterogeneity in Disease

There is no doubt that neutrophils contribute vital function in the development of disease with an indispensable role in innate immunity. It is becoming obvious that neutrophils are much more than microbe-killing cells in diseases, and they display various phenotypes and perform a wide range of functions (Table 1).

### 8.1. Autoimmune Disease

Considerable research has provided evidence for the critical and profound role of neutrophils in promoting the development of autoimmune disease [81]. Those neutrophil functions include, but are not limited to, the following mechanisms: secretion of various cytokines and chemokines; formation of NETs that promote the production of antibodies to citrullinated protein antigens (ACPA) in rheumatoid arthritis or ds DNA in lupus; increased expression of inflammation-related membrane molecules; interactions between cells, including activation of natural killer cells; and release of ROS (reactive oxygen species) and proteases to regulate the release of cytokines [82]. Representative autoimmune diseases are presented below and a discussion of all diseases where neutrophils are known to play a role is impractical and beyond the intended scope of the review.

#### 8.1.1. Rheumatoid Arthritis

Rheumatoid arthritis (RA) is a systemic autoimmune disease characterized by synovial inflammation and cartilage erosion. The view has been expounded that activated neutrophils assemble in inflamed joint tissues, synovial fluid, and involved skin tissues to aggravate the development of RA [83–86]. Circulating neutrophils isolated from RA patients' peripheral blood are functionally different from those in healthy people, being primed for immediate ROS release. First, there are some autologous alteration in RA neutrophils themselves. For example, transcriptional changes take place with high-level expression of TNF [87] and myeloblastin [88], and the expression of membrane-bound receptor activator of NF-*κ*B (nuclear factor *κ*B) ligand (RANKL) is also upregulated in synovial fluid neutrophils [89, 90]. Microenvironmental factors also contribute to strengthen the specific function of RA neutrophils. In the synovial cavity, cytokines like TNF-*α*, IL-8, and granulocyte-macrophage colony-stimulating factor (GM-CSF) [91, 92] help delay the apoptosis of neutrophils and promote neutrophil activation and release of granules [93]. As a result, these stimulated neutrophils secrete various kinds of cytokines and chemokines such as RANKL and BAFF (B cell-activating factor) to activate osteoclasts and B lymphocytes [89, 94] and upregulate the transcription of major histocompatibility complex (MHC) II molecules [95], which may contribute to CD4^+^ T cell activation. All these changes contribute to initiating the advancement of inflammation. In addition, neutrophils at the pannus cartilage junction aggravate matrix degradation through the secretion of MMP-8, MMP-9, neutrophil elastase, cathepsin G, and proteinase 3 [96], all of which have a significant link to cartilage damage in RA. The granule proteins, such as collagenase, gelatinase, and elastase, in RA neutrophils were found in high concentrations and are largely responsible for cartilage and tissue damage [82]. The high concentration of synovial fluid calgranulins induces protease release from specific and gelatinase granules, as well as secretory vesicles [97].

To date, the synovial neutrophils display activating surface markers as CD11b/CD18, CD43, CD63, CD35, CD55, CD66, and CD45 [98–101]. Even though the RA neutrophils are unique for delayed apoptosis, increased ROS release, and intensified intracellular transport, there have been no straightforward evidence or special surface markers for RA-related neutrophils to identify them as a distinct or unique subtype. Despite that, it is conclusive that RA neutrophils have a distinct phenotype compared with neutrophils in other functional states.

#### 8.1.2. Systemic Lupus Erythematosus

Systemic lupus erythematosus (SLE) is considered to be a disease with defects in both innate and adaptive immune regulation [102], which is characterized by the production of autoantibodies to nuclear antigens and immune complex-induced chronic inflammation [103]. Evidence over the last few years have implicated an influential role for neutrophils in the pathogenesis of SLE. Lupus neutrophils display impaired phagocytic capabilities and reduced ability to be removed by the C1q/calreticulin/CD91 pathway [104] as well as abnormal oxidative activity [105–107]. In contrast with RA neutrophils, there are increased numbers of apoptotic neutrophils in SLE, which are related to disease activity and serum levels of anti-double-stranded DNA (anti-dsDNA) antibody. Anti-dsDNA and anti-SS/B antibodies can modulate neutrophil cell death and function [108]. The phenotypic characteristics of SLE neutrophils include enhanced apoptosis, secondary necrosis, and impaired phagocytic capabilities. Neutrophils in SLE also have enhanced NET formation and impaired NET degradation (discussed later).

A distinct subpopulation of neutrophils in SLE is the low-density granulocytes (LDGs) that have attracted much attention in recent years. Although LDGs have been well researched in various autoimmune diseases including idiopathic arthritis [109] and antineutrophil cytoplasmic antibody-associated (ANCA) vasculitis [110] and infections including tuberculosis [111], this review will focus on LDGs in SLE. Lupus LDGs are mononuclear, not polymorphonuclear, cells and included in almost all stages of granulocyte development. They have an enhanced capacity to synthesize proinflammatory cytokines such as interferon gamma (IFN-*γ*) [112, 113]. FACS (fluorescence-activated cell sorting) distinguished LDGs from monocytes by their high expression of CD15 and low expression of CD14, compared to monocytes. In addition, LDGs express CD10, CD16, CD31, CD11c, G-CSFR, and GM-CSFR. Activated lupus LDGs also have surface molecules of CD11b and CD66b [85, 113]. The most prominent characteristic of LDG is similarity in surface markers with a mature neutrophil phenotype (e.g., CD10) but differ the common neutrophil phenotype in the nuclear morphology, which tends to show an immature neutrophil nuclear phenotype. Gene array studies have shown higher mRNA levels of various immune-stimulatory bactericidal proteins and alarmins present in azurophilic granules in the LDGs [112]. Considering that the levels of mRNAs encoding neutrophil serine proteases are highest at the promyelocytic stage in the bone marrow and reduced as granulocytes mature, some investigators hold the view that lupus LDGs are actually immature neutrophils [114]. Since LDGs have both “old” surface markers and “young” nucleus, it is worthy to consider whether lupus LDGs constitute a new subset of neutrophils, or they are just activated cells with distinct phenotype and function. As for their function, LDGs secrete high levels of proinflammatory cytokines including TNF-*α*, IL-6, IL-8, and type I and II IFNs to set up inflammation in lupus. Midgley and Beresford also reported that increased numbers of LDGs in SLE were positively correlated with disease activity and anti-dsDNA antibody level in juvenile SLE (JSLE) [115]. Intriguingly, LDGs are demonstrated to have an effect on cardiovascular incidents in SLE patients. Noncalcified plaque burden (NCB) in SLE patients is positively associated with LDGs, and activated LDGs might contribute to vascular damage and unstable coronary plaque [116].

#### 8.1.3. Multiple Sclerosis

Multiple sclerosis (MS) is characterized by autoimmune inflammation and demyelinating disease. Naegele et al. verified that neutrophils in MS patients had a distinct phenotype with high expression of TLR-2, fMLP (N-formyl-methionyl-leucyl-phenylalanine) receptor, IL-8 receptor, and CD43, as well as displaying a primed state based on reduced apoptosis, stronger degranulation, oxidative burst, and higher levels of NETs in serum [117]. The phenotypic changes might be associated with the specific function of MS neutrophils, and the chronic inflammatory environment in MS may contribute to the active phenotype, thus verifying the elasticity of neutrophils in different states and inflammatory environments.

The experimental autoimmune encephalomyelitis (EAE) model is usually used to study MS. A cluster of extravascular ICAM1^+^ neutrophils in the central nervous system (CNS) in EAE has been demonstrated to gain macrophage-like properties after extravasation. These neutrophils play a role in autoimmune demyelination [118]. They may support inflammation via the enzyme aspartic peptidase retroviral-like 1 (ASPRV1, also known as SASPase), which is, as demonstrated, only expressed by neutrophils in the immune and nervous systems and is necessary for the transition from acute to chronic inflammation in EAE [118]. The expression of ICAM1 distinguishes extravascular macrophage-like neutrophils in EAE, and ICAM1^+^ and ICAM1^–^ neutrophils are differentially distributed in the spinal cord, which again illustrates neutrophil heterogeneity and plasticity.

#### 8.1.4. Diabetes Mellitus

Diabetes mellitus (DM) is a prototypic dysmetabolic syndrome characterized by chronic hyperglycemia and remains a serious health burden globally. Hou et al. used a rapid microfluidic sorting analysis to isolate a subset of rolling neutrophils from peripheral blood of DM patients. This high-rolling-speed phenotype of neutrophil was clarified to have significant correlation with neutrophil activation, rolling ligand P-selectin glycoprotein ligand 1 (PSGL-1) expression, and cardiovascular risk factors associated with DM [119]. Morphologic changes were also detected with a higher number of elongated cells in this high-rolling-speed neutrophil group. A conclusion can be deduced that phenotypic changes in DM patients lead to impaired initial neutrophil capture and rolling, which result in dysfunction in neutrophil-endothelial interaction.

### 8.2. Cancer

Neutrophils have a vital and controversial role in the development of cancer. Circulating PMNs in patients and experimental animals with cancer can be divided into at least three groups according to their density: high-density neutrophils (HDNs), low-density neutrophils (LDNs), which display a segmented nuclear, mature morphology, and granulocytic myeloid-derived suppressor cells (G-MDSCs) with immature morphology [120], and CD14^−^/CD11b^+^/CD15^+^/CD66b^+^/HLA-DR^−^/CD33^+^ cell surface phenotype [121]. LDNs and HDNs commonly show distinct levels of CD11b^+^ expression. A group of sediment granulocytes from renal cell carcinoma patients, which share the same density as PBMC, has higher levels of CD11b^+^ and CD66b^+^ compared to HDNs. LDNs were less segmented than the normal-density PMNs although both of them express membrane markers of CD11b^+^ and CD66b^+^ [122]. MDSCs, on the other hand, are much more heterogeneous among different individuals and can be separated into three groups as CD16^+^/CD11b^+^, CD16^−^/CD11b^+^, and CD16^−^/Cd11b^−^ [123]. The number of circulating neutrophils is increased in both tumor-bearing mouse models and patients with tumor progression [124]. A general mechanism for this phenomenon could be that cytokines produced within the tumor induce the release of G-CSF [125], IL-1, and IL-6 [126]. Tumor-infiltrating neutrophils are considered an independent prognostic factor in tumor recurrence [127–129].

Tissue tumor-associated neutrophils (TAN), traditionally divided into N1 and N2 neutrophils, share a similar surface phenotype with circulating neutrophils including CD66b^+^, CD15^+^, CD16^+^, CD11b^+^, HLA-DR^−^, and arginase-1^+^ (Arg-1). Recent research indicates that the function of TANs varies in different disease states [130, 131]. To be more specific, N1 neutrophils have antitumor function, whereas N2 neutrophils support tumor progression [131]. Since these findings are mostly reported in murine models [124], the nature and biological function of N1 and N2 phenotypes in tumor immunity and progression need better understanding in humans.

The transition of the TAN phenotype and neutrophil dysfunction are strongly influenced by endogenous cytokines released in the tumor microenvironment. Zou et al. [132] found increased numbers of neutrophils in peripheral blood, enhanced tumor infiltration by TANs, and a N2 phenotype transition of infiltrating neutrophils in vivo. These changes in neutrophil number and phenotype were induced by IL-35, which has a high expression in tumor issue. This process is initiated by IL-35-induced production of IL-1*β* and IL-17. IL-17, serving as a protumorigenic factor, is capable of upregulating the expression of IL-6 and G-CSF as well as energizing the recruitment of neutrophils into the tumor immune microenvironment [120]. IL-35 also downregulates TNF-related apoptosis-inducing ligand (TRAIL) expression to enhance the proangiogenic function of neutrophils [132], strengthening tumor growth and disease progression. So, a conclusion can be drawn that the N2 phenotype of TANs in cancer is, at least in part if not wholly, a consequence of IL-35 production by not only tumor cells but also stroma cells and immune cells [133, 134]. Tumor-associated inflammation of neutrophils is also modulated by NK cells. NK cells might act as inhibitors of vascular endothelial growth factor-A (VEGF-A) expression by neutrophils via an IFN-*γ*-stimulated pathway that promotes angiogenesis and, consequently, tumor growth. A higher production of TGF-*β* was also observed in NK cell–depleted tumors, given that TGF-*β* would promote the tumorigenic N2 phenotype [135]. Distant regulation was also found. A unique subset of tumor-infiltrating SiglecF^high^ (Sialic acid-binding immunoglobulin-type lectin) neutrophils was verified to display cancer-promoting properties. The SiglecF^high^ tumor-infiltrating neutrophils in lung cancer are sustained remotely by bone-resident osteocalcin-expressing (Ocn^+^) osteoblastic cells. This group of neutrophils is considered to be effector cells in osteoblast-driven protumoral responses [136].

The interesting function of TANs to facilitate metastasis formation has been well studied. A research of melanoma revealed that tumor cells synthesized IL-8 to upregulate the expression of *β*2 integrin on neutrophils, thus enhancing the neutrophil–melanoma cells' interaction with ICAM-1. This function accelerated the transmigration of melanoma cells through the endothelium. IL-8 also contributes to neutrophil retention in the lung tissue [137]. Additionally, TANs may support the survival and dissemination of tumor cells. CD11b^+^/Ly6G^+^ TANs improve intraluminal survival of tumor cells by inhibiting NK cell function. In addition, the secretion of IL1*β* and matrix metalloproteinases from neutrophils enhances the extravasation of tumor cells [138]. It seems that CD11b^+^/Ly6G^+^ TANs in metastases tend to be N2 phenotype. But intriguingly, this effect can be reversed by TGF-*β* blockade in the tumor microenvironment, which induces CD11b^+^/Ly6G^+^ neutrophils to display an antitumor phenotype [131]. These findings indicate that the phenotype of TAN may be altered artificially from N2 to N1, depending on the tumor microenvironment, thus manifesting the remarkable flexibility and heterogeneity of neutrophils.

CD66b is a very important molecule among TAN surface markers. Huang et al. [139] clarified that CD66b^+^ TANs are significantly increased in number in gastric cancer (GC) and are independently associated with GC prognosis. CD163^+^ TAMs (tumor-associated macrophages) combined with CD66b^+^ TANs could serve as a precise marker to predict the prognosis of GC patients. CD66b^+^ TANs are also found in hepatocellular carcinoma (HCC). They exhibit high expression of programmed cell-death ligand 1 (PDL1), IL8, TNF*α*, and CCL2 but a low expression of CD62L. The upregulation of PDL1 could be a key molecule to maintain the survival and function of activated TANs through an IL6-STAT3-PDL1 signaling cascade driven by cancer-associated fibroblasts (CAFs) [140]. Increased survival rate in colorectal cancer is also associated with CD66b^+^ TAN infiltration, and these neutrophils enhance the activation of CD8^+^ T cells in cocultural system [141], which might function as antitumor cells and have a positive effect on the prognosis of patients.

Much detail of TAN functions has been derived from research in animal models, but research on the function of human TANs is controversial. There have been two controversial reports on human TAN's immunosuppressive capacity. Eruslanov et al. [142] isolated TANs from digested human lung tumors. These TANs displayed a phenotype of CD62L^low^CD54^hi^ and produced a number of proinflammatory cytokines that enhanced T cell proliferation and IFN-*γ* production. Wu et al. [143], on the other hand, collected TANs from colorectal tumor tissue that presented a phenotype of CD45^+^Lin^−^HLADR^−^CD11b^+^CD33^+^CD66b^+^, which was similar to the classical neutrophil morphology. Those TANs produced arginase 1 and ROS and downregulated T cell proliferation and IFN-*γ* production. One possible explanation for the different results could be that different tumor microenvironments may determine the TAN's heterogeneity in both phenotype and function.

Although neutrophils have historically not been considered as MHC class II APC, they are now well established as being able to express MHC class II under certain conditions and function as “atypical” APC [144]. Yuan et al. [145] clarified that CXCL1-induced, tumor-infiltrated neutrophils have increased expression of MPO (myeloperoxidase) and Fas/FasL (also known as CD95/CD95L), which may be involved in TAN-mediated inhibition of CD4^+^ and CD8^+^ T cells. TANs displaying characteristics of both neutrophils and antigen-presenting cells (APCs) were identified in early-stage human lung cancer [146]. These hybrid neutrophils defined as CD11b^+^Arg-1^+^CD66b^+^CD15^+^HLA-DR^+^CD14^+^ have the capacity to trigger antitumor T cell responses as well as cross-present antigens, which builds an intriguing connection between innate and adaptive immunity. Cohort studies on TAN phenotypes and functions have generated considerable phenotypic data on TAN in different human cancers. Changes to neutrophil membrane markers in childhood acute lymphoblastic leukemia (ALL) were reported in a 118 BCP-ALL cohort study [147]. Around 77% of the cases showed altered markers, including CD10 (53%), CD33 (34%), CD13 (15%), CD15/CD65 (10%), and CD123 (7%), although no correlation was found between altered markers and clinical features. So, the biological relevance of the abnormal phenotypes has yet to be resolved. Another cohort study [148] in head and neck squamous cell carcinoma (HNSCC) patients verified the antitumor function of CD16^high^CD62L^dim^ neutrophils. The CD16^high^CD62L^dim^ neutrophils inhibited migration and proliferation and induced apoptosis of cancer cells via NET formation. An increased proportion of CD16^high^CD62L^dim^ neutrophils was correlated with increased survival rate in that cohort. The role of neutrophils, particularly tumor-infiltrating neutrophils, in inhibiting or promoting tumor growth and the variation in phenotypes associated with the disparate functions, is emphasis of the remarkable heterogeneity and plasticity of neutrophils.

### 8.3. Inflammation

As the vanguard of immune cells in sites of inflammation, it is not surprising for neutrophils to switch into different phenotypes with multiple functions during infection and inflammation. Specific functions are usually associated with altered phenotypes, thus defining prominent functional subpopulations adapted to the microenvironment and characteristics of relevant innate or adaptive immune stimuli.

Infection and inflammation related neutrophils show quite different characteristics with those in other diseases and may have great heterogeneity even in the same microenvironment. In the condition of methicillin-resistant *Staphylococcus aureus* (MRSA) infection, two distinct subsets of neutrophils named PMN-1 and PMN-2 were first reported in 2004 [149]. PMN-1 neutrophils were characterized as CD49d^high^CD11b^low^ with upregulated expression of TLR2, TLR4, TLR5, and TLR8, while PMN-2 cells were CD49d^low^CD11b^high^ with upregulated TLR2, TLR4, TLR7, and TLR9. There was also functional heterogeneity in cytokine secretion between PMN-1 and PMN-2. PMN-1 were prone to IL-2 production while PMN-2 were prone to IL-10. Suppression of PMN-2 or enhancement of PMN-1 led to the protection of immunocompromised hosts against MRSA infection.

Gout is an acute inflammatory disease mostly presenting with symptoms of joint swelling, redness, and pain attributed to the precipitation of uric acid in the form of monosodium urate (MSU) crystals [150] that stimulate inflammation mainly in joints. MSU crystals stimulate a NALP3 inflammasome-dependent acute inflammation [134]. Numerous chemokines are released into affected, inflamed sites, such as CXCR2, CXCL-8, CXCL-1, CXCL-2, and CXCL-3 [151], inducing accumulation of neutrophils and release and aggregation of NETs. The role that NETs play in gout seems to be protective. MSU crystals are embedded within NET chromatin, and proteins from granules including serine proteases associated with NETs help degrade inflammatory cytokines [152]. NETs also have an anti-inflammatory function by blocking further accumulation of neutrophils through lactoferrin synthesis [153]. MSU-induced NETs are enriched for actin and are less sensitive to DNase degradation [154]. Distinctly, NETs contribute to the resolution of inflammation in gout.

Only a few neutrophils are maintained in the lung in normal homeostatic conditions. Bronchiectasis is a predominantly neutrophilic condition. Circulating neutrophils in bronchiectasis have a significantly prolonged lifespan, delayed apoptosis, increased CD62L shedding, upregulated CD11b expression, increased myeloperoxidase release, and impaired phagocytosis and killing of *Pseudomonas aeruginosa* (PAO1) that is associated with a worse outcome [155]. These aberrant functional activities of neutrophils in bronchiectasis indicate an attenuated capacity for bacterial elimination. Neutrophils overexpressing ICAM-1 had enhanced effector functions including phagocytosis and reactive oxygen species (ROS) generation [156]. An increased number of ICAM-1^+^ neutrophils were shown to accumulate in the lung tissue during sepsis, leading to acute respiratory distress syndrome (ARDS). The abnormal accumulation of ICAM-1^+^ neutrophils was related to cold-inducible RNA-binding protein (CIRP), a damage-associated molecular pattern (DAMP) [157]. CD49d^+^ CysLTR1^+^ (cysteinyl leukotriene receptor 1) neutrophils isolated during acute viral respiratory tract infection produced TNF, CCL2, and CCL5 and were necessary for the complete development of postviral atopic disease [158]. However, further research is needed to validate the function of this group of neutrophils. CEACAM6^high^ (CEACM6, nonspecific cross-reacting antigen, also known as CD66c) neutrophils were isolated from bronchi in severe asthma patients and were considered to be a vital biological feature of treatment-resistant severe asthma [159]. The expression of CEACAM6 protein was upregulated in both bronchi epithelial cells and lamina propria neutrophils in patients with severe asthma. Homophilic binding of CEACAM6 to N-domain CEACAM6 peptides could potentially enhance neutrophil activation with the generation of superoxide [158] potentially contributing to neutrophil activation and epithelial damage as well as respiratory dysfunction in asthma [155].

Circulating neutrophils from visceral leishmaniasis patients have been shown to have reduced CXCL8 expression but increased IL-10 and arginase-1 expression with enhanced capacity to phagocytose *Leishmania* spp. promastigotes [160]. These functions may support an immunosuppressive role of neutrophils in active visceral leishmaniasis, but prominent phenotypic markers for this subset of neutrophils are unknown.

In a cohort liver cirrhotic study in 2016 [161], EMR2^high^ (EGF-like molecule containing mucin-like hormone receptor 2) neutrophils were verified to be associated with disease severity and to predict the rate of mortality in cirrhotic patients. These EMR2-expressing neutrophils displayed an activated phenotype with a higher-level expression of activation molecules such as CD11b, CD181, CD182, and CD49d, but these neutrophils also showed deranged function including increased resting oxidative burst and impaired phagocytosis ability. Besides, EMR2^high^ neutrophils were also correlated with higher mortalities in cirrhotic patients. Thus, these neutrophils can be considered as a significant parameter to predict the outcome of liver cirrhosis in patients.

## 9. Neutrophils and NETs

An alternative pathway for the death of neutrophils besides necrosis or apoptosis is the formation of NETs. NETs are extracellular strands of unwound chromatin in complex granule proteins including those with inflammatory and bactericidal activity. The NET granule proteins include neutrophil elastase (NE), myeloperoxidase (MPO), cathepsin G, lactoferrin, pentraxin 3, gelatinase, proteinase 3, peptidoglycan-binding proteins, and DNA-free histones [162]. There are at least 24 neutrophil proteins associated with NET formation according to mass spectrometry analysis [163]. Classical NET formation pathways include activation of integrins and Toll-like receptors (TLR) in response to bacterial-associated pathogen-associated molecular patterns (PAMPs) [164]. The prominent mechanism of NET formation is dependent on ROS and the Raf/MERK/ERK (rapidly accelerated fibrosarcoma (Raf)/mitogen-activated protein kinase ERK kinase (MERK)/extracellular signal-regulated kinase (ERK)) pathway. After neutrophil activation, nicotinamide adenine dinucleotide phosphate oxidase (NOX) increases via protein kinase C (PKC), resulting in cytosolic calcium intake, peptidyl arginase deaminase 4 (PAD4) activation, and chromatin decondensation. When cytosolic calcium increases, PAD4 activation and chromatin decondensation occur [165]. Secondly, ROS promotes the loss of the nuclear membrane. Then chromatin spreads throughout the cytoplasm together with cytoplasmic and granule proteins and finally NETs are released out of the cell. Another mechanism independent of ROS and the Raf/MERK/ERK pathway evolves through three stages: nuclear envelope growth and vesicle release, nuclear decondensation, and nuclear envelope disruption [166]. The NETosis, although not unique to neutrophils, is *bona fide* a well-recognized and important phenomenon in neutrophil function, so this section will discuss NETs and NETosis in detail in diseases where they are recognized as particularly important.

### 9.1. NETs in Inflammation and Infection

It is generally considered that NET formation plays a positive role in attenuating inflammation during infection since neutrophils primed by microbiota are more prone to form NETs [26]. A UK cohort study [167] identified neutrophil dysfunction in sepsis that included significantly decreased NET formation accompanied by defects in neutrophil migration and delayed apoptosis. These abnormal changes forebode poor outcomes with increased 30-day and 90-day mortality. In addition, NETs appeared to display a positive role in *Streptococcus suis* serotype 2 (SS2) infection. Although SS2 biofilms are capable of inhibiting NET formation, NETs derived from neutrophils stimulated by planktonic bacteria and host inflammatory factors still display the ability to eliminate bacterial biofilms [168]. This research elucidates a novel view on the battles between NETs and bacterial biofilms. In the examples of bacterial infection cited above, NETs have a protective function. In other types of infection, NETs may have a deleterious effect. NET formation was associated with *Sendai virus* (Sev) infection and amplified early inflammation in the lung in the Sev-induced asthma model, consequently priming an inflammatory cascade, immune cell activation, and airway remodeling [169]. Neutrophil-derived cysteine protease dipeptidyl peptidase I (DPPI) is an important mediator in NET formation, and inflammatory conditions such as sepsis were less severe in the absence of DPPI [170]. To summarize, the role of NETs in infection and inflammation is variable and dependent upon the activating stimulus.

Findings described above identify the value of NETs as potential new biomarkers of disease activity, prognosis, and NETosis as potential therapeutic targets. Future studies are needed to illustrate and understand the detailed mechanisms and relationships between NETs and diseases.

### 9.2. NETs in Autoimmune Disease

The function of NETs in autoimmunity has been reviewed recently [133]. Externalized DNA is a DAMP with potent innate immune-activating potential, and it is more resistant to degradation [171]. The persistence of nuclear components encompassed in NETs, particularly DNA, creates a potent source of autoantigens. Apoptotic neutrophils in the circulation could also be a source of autoantigens in SLE patients. These autoantigens such as dsDNA and cathelicidin (LL-37) show an increased level in peripheral blood and correlate with disease activity in SLE patients [172]. NETs released from LDGs displayed high levels of autoantigens and immune-stimulating molecules, including MMP-9, *α*- and *β*-defensins, and LL-37 [112], and the ability for SLE patients to clear NETs is impaired [173]. A high level of type I IFN, which enhances autoimmune B cell activation, is a critical feature in SLE [174, 175] and is correlated with demethylated CpG DNA, LL-37, and other NET contents, all of which upregulate type I IFN expression by plasmacytoid dendritic cells (pDC) [176]. These processes initiate a progressive cycle to induce more NETs that stimulate more type I IFN production and exacerbated chronic inflammation and B cell activation and autoantibody production. The inhibition of FcgRII, NADPH (nicotinamide adenine dinucleotide phosphate) oxidase, or TLR7 are inhibitors of NETs [177]. All these findings have provided new insights into the role of neutrophils and NETosis in the generation of the type I IFN signature in SLE. As such, neutrophils and NETosis are potential targets for future therapeutics.

### 9.3. NETs in Cancer

NETs are *per se* a double-edged sword. NETs could induce the proliferation and malignant transformation of B cells toward malignant lymphoma via NF-*κ*B signaling [178], and in addition, some NET-induced cytokines reveal a relationship between NETs and tumor development. IL-8 has been demonstrated to play a role in both NET generation and angiogenesis as well as tumor progression [179, 180], and granulocyte colony-stimulating growth factor (G-CSF) was also demonstrated to be associated with tumor generation [181]. Notably, NETs may play a crucial role in hematogenous metastasis. It has been verified that metastatic breast cancer cells induced neutrophils to form NETs, which enhanced tumor cell growth in target organs [182]. Kanamaru et al. found that CD66^+^ mature LDNs were observed to cluster in the peritoneal cavity within patients who underwent laparotomy due to gastric cancer. These LDNs released NETs with the typical features of threadlike structures positive for nucleic acid staining, histones, and myeloperoxidase [183]. *In vitro* experiments indicated that tumor cells attached to NETs did not die but continued to proliferate. Additionally, it was verified that NETs helped upregulate the level of MMP-9 to degrade extracellular matrix, which facilitated tumor invasion [1]. These findings support the conclusion that NETs may enhance the clustering and growth of free tumor cells.

Recently, intriguing discoveries of an association between cancer thrombosis and NETs have drawn attention. It has been reported that NETosis was associated with microcirculatory thrombosis [184] leading to thromboembolic complications in cancer. Spontaneous intestinal tumorigenesis was verified to correlate with the accumulation of N2 phenotype LDGs, as well as NET formation and hypercoagulation. The potential mechanism was inferred that stimuli such as circulating LPS could upregulate complement C3a receptor on neutrophils. C3aR plays an important role in NETosis [185] since C3a-activated neutrophils become more susceptible to NETosis. This process aggravated thrombus formation, which induced a N2 phenotype in the neutrophils. These neutrophils underwent spontaneous NETosis, further exacerbating hypercoagulation and initiating a progressive cycle [186]. In another way, NETs could cooperate with tumor-derived exosomes to induce the establishment of venous and arterial thrombus formation in breast cancer [187] through the stimulation of platelet aggregation, activation of contact pathways, and degradation of natural coagulation inhibitors [188, 189]. More detailed research on these associated mechanisms is still on the exploratory stage.

## 10. Conclusion

The brand-new discoveries of neutrophil plasticity in various conditions broaden the horizon that neutrophils are not just simple reproductions. They display strong heterogeneity in morphology and function in both healthy and disease circumstances including infection, tumorigenesis, tumor immunity, and autoimmunity. Even though some researchers hold the view that several characteristic classes of neutrophils, such as LDGs and N1/N2 TANs, are *bona fide*, independent subpopulations based upon a host of supporting evidence, others interpret the heterogeneity of form and function as just manifestations of differential activation. Researches have elucidated distinct functions of neutrophils with up- or downregulated membrane molecules in various contexts, but further relation and mechanism that correlate those phenotypic differences are still being revealed. The interaction of phenotype and function in neutrophils will continue to be worthy of attention in the future. There is a reason to consider neutrophils as highly malleable cells and most type features can be acquired at specific sites after stimuli, but the variations occurring in the early immature stage or the late mature stage are still poorly understood. We expect more valuable studies to expand existing recognition of neutrophils' multiple roles.

## Figures and Tables

**Table 1:** Neutrophils' phenotypes and functions in different states.

States	Representative phenotype	Function	Reference
Health			
Neutrophils in circulation	CXCR4^high^CXCR2^high^CD62L^low^	Migration	[21, 23]
Aged neutrophils	CD11b^+^CD49d^+^Mac-1^+^VLA-4^+^ICAM-1^+^TLR4^+^CD47^low^	Migration Anti-inflammation Resistance to phagocytosis	[26–28]
Tissue-resident neutrophils			
Lung	*β*2 integrins, VLA4^+^CXCR4^+^	Adhesion Transmigration Deformation	[1, 22, 50]
Spleen	CD62L^low^ CD11b^hi^ ICAM-1^hi^, CD11b^+^, CXCR4^+^, CCR7^+^	Migration Activated apoptosis	[51, 52, 54, 55]
Disease			
Autoimmune disease			
RA	CD11b/CD18^+^CD43^+^CD63^+^CD35^+^CD55^+^CD45^+^CD66^+^	Complement regulation Adhesion Inflammatory activation	[88–96, 98–101]
SLE	LDGs:CD15^+^CD14^low^CD10^+^CD16^+^CD31^+^CD11c^+^G-CSFR^+^GM-CSFR^+^	Inflammatory activation Activated apoptosis Correlated with disease activity	[85, 109–113]
MS	TLR2^+^fMLPR^+^IL-8R^+^CD43^+^	Activated apoptosis	[117]
EAE	ICAM-1^+^	Autoimmune demyelination	[118]
DM	PSGL-1^+^	Dysfunction in neutrophil-endothelial interaction	[119]
Cancer			
Typical clusters			
G-MDSC	CD14^−^CD11b^+^CD15^+^CD66b^+^HLA-DR^−^CD33^+^	—	[118, 119]
TAN	CD66b^+^CD15^+^CD16^+^CD11b^+^HLA-DR^−^ Arg-1^+^	N1: antitumoral function N2: support tumor progression	[130, 131]
Melanoma	High expression of *β*2 integrins	Transmigration N2 activation	[137]
Metastases	CD11b^+^Ly6G^+^	N2 activation	[138]
GC	CD66b^+^	Associated with GC prognosis	[140]
HCC	CD66b^+^	Proinflammatory activation	[141]
Lung tumor	CD62L^low^ CD54^high^	N1 activation	[142]
Colorectal tumor	CD45^+^Lin^−^HLA-DR^−^CD11b^+^CD33^+^ CD66b^+^	N2 activation	[143]
ALL	Altered expression of CD10, CD33, CD13, CD15/CD65, and CD123	No correlation with clinical features	[147]
HNSCC	CD16^high^CD62L^dim^	N1 activation Correlated with increased survival rate	[148]
Infection/inflammation			
MRSA			
PMN-1	CD49d^high^CD11b^low^ TLR2^high^TLR4^high^TLR5^high^TLR8^high^	Protective activation IL-2 producing	[149]
PMN-2	CD49d^low^CD11b^high^ TLR2^high^TLR4^high^TLR7^high^TLR9^high^	Aggravating infection IL-10 producing	[149]
Gout	—	Activated apoptosis	[151–154]
Bronchiectasis	CD11b^high^CD62L^high^	Inflammatory activation Impaired phagocytosis	[155, 156]
Sepsis	ICAM-1^+^	Accumulation and migration Inflammatory activation	[157]
Acute viral respiratory tract infection	CD49d^+^CysLTR1^+^	Further researches needed	[158]
Asthma	CD66c^high^	A biological feature of treatment-resistant asthma	[155]
Cirrhotic	EMR2^+^CD11b^high^CD181^high^CD182^high^CD49d^high^	Inflammatory activation Impaired phagocytosis; correlated with infectious complications	[158]

RA: rheumatoid arthritis; SLE: systemic lupus erythematosus; MS: multiple sclerosis; EAE: autoimmune encephalomyelitis; DM: diabetes mellitus; G-MDSC: granulocytic myeloid-derived suppressor cells; TAN: tumor-associated neutrophils; GC: gastric cancer; HCC: hepatocellular carcinoma; ALL: acute lymphoblastic leukemia; HNSCC: head and neck squamous cell carcinoma; MRSA: methicillin-resistant *Staphylococcus aureus*.
